# Vitamin B12 Deficiency Alters the Gut Microbiota in a Murine Model of Colitis

**DOI:** 10.3389/fnut.2020.00083

**Published:** 2020-06-05

**Authors:** Eberhard Lurz, Rachael G. Horne, Pekka Määttänen, Richard Y. Wu, Steven R. Botts, Bo Li, Laura Rossi, Kathene C. Johnson-Henry, Agostino Pierro, Michael G. Surette, Philip M. Sherman

**Affiliations:** ^1^Cell Biology Program, Research Institute, Hospital for Sick Children, Toronto, ON, Canada; ^2^Division of Gastroenterology, Hepatology and Nutrition, Department of Paediatrics, Toronto, ON, Canada; ^3^Department of Laboratory Medicine and Pathobiology, University of Toronto, Toronto, ON, Canada; ^4^Physiology and Experimental Medicine, Research Institute, Hospital for Sick Children, Toronto, ON, Canada; ^5^Department of Medicine, Department of Biochemistry and Biomedical Sciences, McMaster University, Hamilton, ON, Canada; ^6^Division of General and Thoracic Surgery, Hospital for Sick Children, Toronto, ON, Canada; ^7^Farncombe Family Digestive Health Institute, McMaster University, Hamilton, ON, Canada

**Keywords:** vitamin B12, inflammation, microbiome, inflammatory bowel disease, colitis

## Abstract

**Purpose:** Inflammatory bowel disease (IBD) refers to a spectrum of autoimmune diseases, which result in chronic intestinal inflammation. Previous findings suggest a role for diet, nutrition and dysbiosis of the gut microbiota in both the development and progression of the condition. Vitamin B12 is a key cofactor of methionine synthase and is produced solely by microbes. Previous work links increased levels of homocysteine, a substrate of methionine synthase, MetH, to IBD indicating a potential role for vitamin B12 deficiency in intestinal injury and inflammation. This study assessed the role of vitamin B12 in shaping the gut microbiota and determining responses to intestinal injury using a reproducible murine model of colitis.

**Methods:** The effects of vitamin B12 supplementation and deficiency were assessed *in vivo*; 3-week-old post-weanling C57Bl/6 mice were divided into three dietary treatment groups: (1) sufficient vitamin B12 (50 mg/Kg), (2) deficient vitamin B12 (0 mg/Kg) and (3) supplemented vitamin B12 (200 mg/Kg) for a period of 4 weeks. Intestinal injury was induced with 2% dextran sodium sulphate (DSS) via drinking water for 5 days. The impact of varying levels of dietary vitamin B12 on gut microbiota composition was assessed using 16S rRNA gene sequencing from fecal samples collected at day 0 and day 28 of the dietary intervention, and 7 days following induction of colitis on day 38, when blood and colonic tissues were also collected.

**Results:** No significant alterations were found in the gut microbiota composition of disease-free animals in response to dietary interventions. By contrast, after DSS-induced colitis, >30 genera were significantly altered in vitamin B12 deficient mice. Altered B12 levels produced no significant effect on composite disease-activity scores; however, administration of a B12 deficient diet resulted in reduced DSS-induced epithelial tissue damage.

**Conclusions:** Vitamin B12 supplementation does not alter the gut microbiota composition under healthy conditions, but does contribute to differential microbial responses and intestinal dysbiosis following the induction of experimental colitis.

## Introduction

Inflammatory Bowel Disease (IBD) encompasses a spectrum of intestinal diseases including ulcerative colitis and Crohn's disease (1) and is characterized by chronic, relapsing and remitting mucosal inflammation in the intestinal tract (2). Increasing evidence associates the development of IBD with reduced microbial diversity of the gut microbiota (3) and dietary alterations (4) in a genetically susceptible host (5). While the direct cause for IBD development is still not fully understood, micronutrient deficiencies are commonly associated with IBD. Within this population under-nutrition is frequently associated with both inadequate dietary intake and inadequate absorption due to underlying intestinal disease activity. Therefore, micronutrient deficiencies have been hypothesized to play a role in either the development or the progression of IBD (6).

Vitamin B12, also known as cobalamin, acts as a coenzyme of methionine synthase which catalyzes the conversion of homocysteine to methionine (7). Vitamin B12 deficiency leads to hyperhomocysteinemia characterized by high levels of homocysteine (8). Hyperhomocystiene is an established risk factor for cardiovascular disease and is associated with arterial wall stiffness (9) which is often increased in patients with IBD (10). An association of hyperhomocysteinemia with IBD has also been previously described (11), with increased homocysteine levels present in gut tissues of IBD patients (both Crohn's disease and ulcerative colitis), compared to intestinal mucosa taken from healthy controls (12). Hyperhomocysteinemia can also result from folate deficiency, as both folate and vitamin B12 are important cofactors of methionine synthase (13).

To date, there are conflicting results about the effects of vitamin B12 status on intestinal inflammation, and no studies have investigated whether alterations in the composition of the gut microbiome are associated with vitamin B12 deficiency. Benight et al. (14) reported that vitamin B12 deficiency protects against DSS-induced colitis in mice. By contrast, Bressenot et al. (15) noted reduced intestinal barrier function in vitamin B12 deficient rats. A third study found variations in the effects of acute cobalamin administration in a murine model of colitis based on its bioactive state: methylcobalamin having more anti-inflammatory properties, whereas synthetic cyanocobalamin had a more pro-inflammatory effect (16).

The gastrointestinal tract is the primary site of disease in IBD, with specific regions affected differentially based on disease subtype. The gastrointestinal tract is also home to trillions of microorganisms that form a complex community referred to as the gut microbiota. The gut microbiota aids in the development of the host immune system, energy metabolism, and defense against enteric pathogens (17–19). The gut microbiome can be altered in a variety of non-communicable, chronic diseases (20), with nutrition having a significant impact on the gut microbiota. Numerous studies indicate that dietary changes can alter both the composition and function of gut microbes (21). In this study, therefore, we assessed the impact of changes in dietary vitamin B12 on the severity of mucosal injury and fecal microbiota composition in a murine model of colitis.

## Materials and Methods

### Animal Model

**Figure 1:**
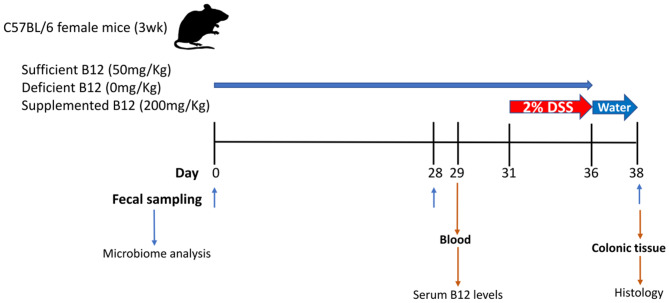
Graphical representation of the experimental design for vitamin B12 administration in a dextran sodium sulphate (DSS)-induced mouse model of colitis. Three-week-old female C57BL/6 mice were provided specific diets containing varying levels of vitamin B12 for 4 weeks. DSS was administered for 5 days followed by 2 days of water alone. Fecal pellets were obtained on days 0, 28, and 38. Blood and colon samples were collected at the time of sacrifice on day 38.

Weaned three-week-old female C57BL/6 mice (Jackson Laboratories, Bar Harbor, ME) were housed in the containment unit of the Laboratory Animal Service facility at the Hospital for Sick Children (Toronto, ON, Canada). All procedures and protocols were adhered to and were approved by the Animal Care Committee at the Hospital for Sick Children (Protocol# 37290). Mice were allowed free access to chow and sterile drinking water during the study protocol (Figure 1). Three Teklad custom diets were purchased from Harlan Laboratories Canada Ltd. (Toronto, ON, Canada). All three diets consisted of a modification of the standard AIN-93G rodent diet with ethanol washed casein to reduce background B12: 1) vitamin B12 sufficient 50 mg/Kg, 2) vitamin B12 deficient 0 mg/Kg, and 3) vitamin B12 supplemented 200 mg/Kg. Vitamin B12 (cyanocobalamin) concentrations in chow were measured by ENVIGO laboratories (Madison, WI) after irradiation using the Official Methods of Analysis, Method 952.20 and 960.46, AOAC INTERNATIONAL (Gaithersburg, MD). All diets were matched for macronutrient composition and energy content (18.3% protein, 60.1% carbohydrate, 7.0% fat by weight, 3.8 Kcal/g).

Mice were divided into a DSS experimental group (*n* = 15) and control group (*n* = 9). Both the DSS experimental group and control group mice were subdivided into 3 diet treatment groups: (1) vitamin B12 sufficient (2) vitamin B12 deficient; (3) vitamin B12 supplemented. The inclusion of a control group with sufficient vitamin B12 levels was included to enable an evaluation in shifts in the gut microbiome in relation to the transition from mother's milk to mouse chow. Mice were weighed and fecal pellets collected on day 0, day 28, and day 38 to assess changes in microbial communities. DSS was administered in drinking water (2% wt/vol, MP Biomedicals, Solon, OH) on day 31 for 5 days to the DSS experimental group only, after which animals received water alone for 2 days (22). Animals were monitored for changes in weight, health, and well-being and variations recorded throughout the experimental procedure.

### Analysis of Vitamin B12 Levels in Serum

Whole blood was obtained from the facial vein on day 29, two days prior to DSS-induction of colitis. Blood samples were centrifuged at 2,300 × g, at 4°C for 20 min, and serum isolated and stored at −20°C. Vitamin B12 levels were measured quantitatively using the Chemiluminescent Microparticle Intrinsic Factor assay (ARCHITECT B12 assay; Abbot Park, IL).

### Evaluation of Epithelial Injury

At the end of the study protocol animals were administered CO_2_ prior to cervical dislocation and colons were excised and lengths measured *ex-corpus*. Segments of distal colon were fixed in 10% neutral-buffered formalin and paraffin embedded. Samples were then sectioned, stained with hematoxylin and eosin (H&E) and visualized using a Leica DMI 6000B microscope equipped with a Leica DFC420 camera (Leica, Dialux 22: Leica Systems, Willowdale, ON, Canada), as described previously (23). Tissue sections were assessed and scored by a blinded individual. Histopathology was graded numerically by disease activity scores (DAS), consisting of the combined scoring of the severity of epithelial tissue injury (graded 0–3, from absent to mild including superficial epithelial injury, moderate including focal erosions, and severe including multifocal erosion), the extent of inflammatory cell infiltrate (graded 0–3, from absent to transmural), and goblet cell depletion (graded 0–2, from no change in goblet cell number to no visible goblet cells), as previously described (24).

### Immunofluorescence

Detection of Muc2 production in colonic tissues was carried out on fixed sections, as previously described (25). Briefly, colonic sections were incubated with primary rabbit anti-Muc2 (Santa-Cruz, Dallas, TX) overnight at 4°C followed by Alexa Fluor 488-conjugated secondary (Invitrogen, Carlsbad, CA) and DAPI (Vector Laboratories, Burlingame, CA) at room temperature for 2 hours. Images were acquired using a Nikon TE-2000 digital microscope equipped with a Hamamatsu C4742-80-12AG camera.

### Reverse Transcriptase qPCR

Full-thickness distal colonic specimens were collected at the time of sacrifice and frozen at −80^o^C. Collected tissues were homogenized and total RNA extracted by Trizol (Invitrogen). Isolated RNA was treated with DNase A (Invitrogen), as per manufacture's guidelines. RNA quality and yield were assessed by A260/A280 and A260/A230 ratios and analyzed with a Nano-Drop® ND-1000 spectrophotometer (NanoDrop Technologies). A total of 1 μg of RNA was transcribed into cDNA, using an iSCRIPT cDNA synthesis kit (Bio-Rad). cDNA was amplified by qPCR, using SsoFast EvaGreen Supermix and a CFX96 C1000 Thermal Cycler (Bio-Rad). Primers against mouse GAPDH and ribosomal protein L10 (RPL10) (housekeeping genes), interleukin-10 (IL10) and tumor necrosis factor α (TNF-α) were used. Primer sequences used in the assay can be found in Supplemental Table 1. The comparative ddCt–method was used to determine the amount of target gene normalized to endogenous references (GAPDH and RPL-10) and relative to a calibrator used in the control group.

### 16S rRNA Gene Analysis

As previously described (26, 27), a FastDNA™ spin kit for soil (MP Biomedicals, Irvine, CA) was used to extract DNA from fecal pellets collected on day 0, day 28, and day 38 from DSS-treated animals as well as the control group on day 38. Sequences of the 16S rRNA gene variable 3 (V3) region were amplified with a previously described procedure (26), with modifications previously described (27, 28), and sequenced using the Illumina MiSeq platform. Sequence data have been submitted to the European Nucleotide Archive (ENA) and can be accessed under study accession PRJEB37474. Operational taxonomic units (OTUs) were picked with AbundantOTU+ (29) with a clustering threshold of 97% sequence similarity. The Ribosomal Database Project (RDP) classifier (30) assigned taxonomy up to the genus level using the Greengenes 2013 reference database (31). Using QIIME version 1.9.1 (32), singleton OTU's were removed and a final OTU table generated for further analyses. The OTU table was rarefied to 12,000 reads prior to beta and alpha diversity analyses, which were completed using the phyloseq and vegan packages in R version 3.5.2 (33), and graphed using ggplot2 and edited in Inkscape (v0.28.1). For genus level statistics, total sum scaling was employed to account for variation in sequence depth with the top 25 most abundance taxa assessed for differential abundance.

### Statistical Analyses

All statistical analyses for 16S rRNA sequencing data were performed in R version 3.5.2 (33). Total sum scaling normalization was performed prior to assessing differences in taxa at the genus level within treatment groups using a two-sided unpaired permutation t-test and corrected for multiple comparison using the Benjamin Hochberg procedure (FDR). Between treatment group analysis of relative abundance was assessed using the Kruskal Wallis test and corrected with FDR. Permutational multivariate analysis of variance (PERMANOVA) was used to assess differences in beta diversity. All other significance testing was performed in Graphpad using either Student's t-test or ANOVA with Tukey's *post-hoc* testing, with *P* < 0.05 considered as statistically significant.

## Results

### Serum Vitamin B12 Levels Increase With Supplementation

**Figure 2:**
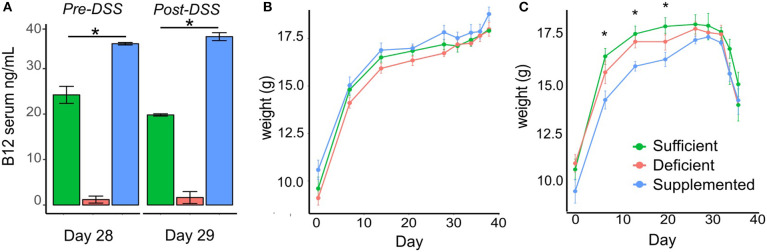
Vitamin B12 levels in serum and the effect of dietary vitamin B12 on weight. **(A)** Serum B12 levels measured at day 29 pre-DSS treatment and post-DSS treatment on day 38 show significant increased levels with supplementation and decreased levels with deficient diets in both control and DSS mice (*P* < 0.0001). **(B-C)** Body weight changes in the three dietary groups from Control **(B)** and DSS **(C)** mice (one-way ANOVA, *p < 0.05, *n* = 5).

Mice fed a vitamin B12 deficient chow exhibited significantly lower vitamin B12 serum levels (1.19 ± 0.58 ng/ml) on day 29 of the study protocol, compared to animals fed either a vitamin sufficient (24.23 ± 1.88 ng/ml) or supplemented (35.48 ± 1.73 ng/ml) diet. Similar trends in serum levels were observed in mice administered deficient (1.64 ± 1.31 ng/ml), sufficient (19.82 ± 0.25 ng/ml), and supplemented (37.04 ± 0.87 ng/ml) diets post DSS treatment (*P* < 0.0001, *n* = 5) (Figure 2A).

### Vitamin B12 Status Does Not Impact DSS-induced Weight Loss

Change in body weight was monitored over the course of the experiment in both the control and experimental DSS treated mice. There were no significant differences in body weight in control animals fed B12 deficient, sufficient or supplemented vitamin B12 chow diets (Figure 2B). In the experimental group designated for DSS treatment, differences in body weight (*p* < 0.04) were observed before DSS administration on day 7, 14, and 21, however these differences were no longer evident by day 28 (Figure 2C).

### Effect of DSS and B12 on Intestinal Pathology

**Figure 3:**
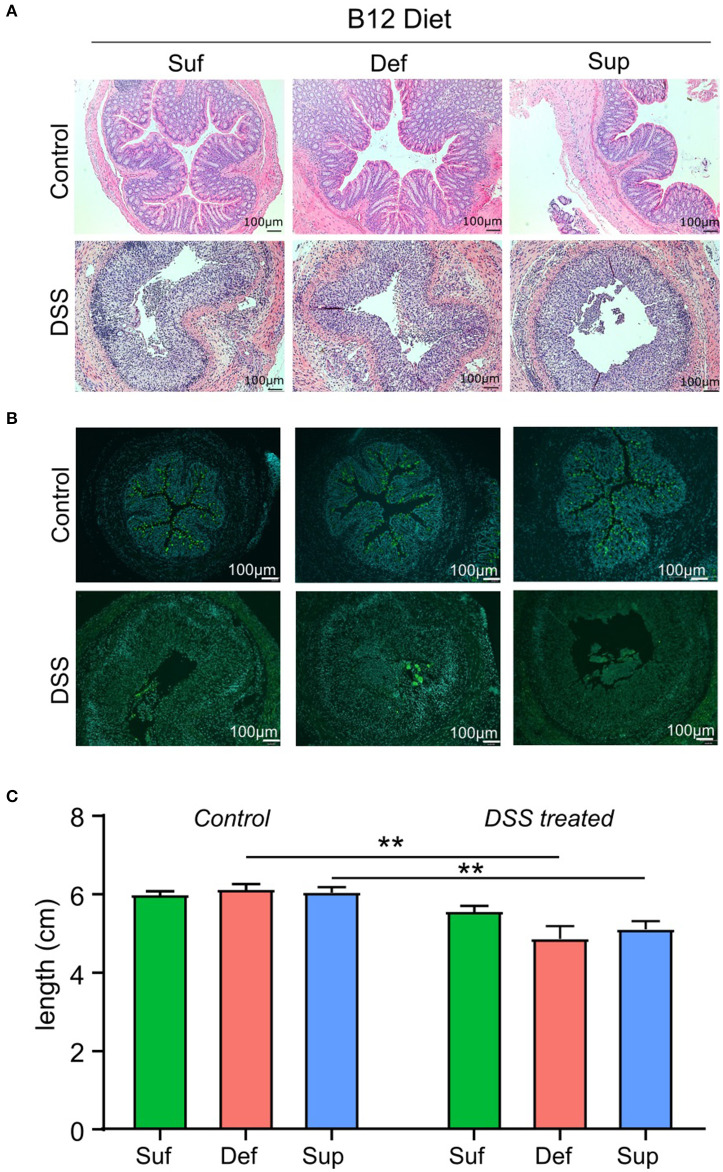
Markers of colonic epithelial injury. **(A)** Representative hematoxylin and eosin staining of colonic sections showing normal morphology in control groups and marked tissue damage. **(B)** Representative images depicting the presence of Muc2 punctate staining in colonic tissue from control mice and loss of Muc2 staining in animals administered DSS and fed either vitamin B12 sufficient or vitamin B12 supplemented diets. **(C)** Colon length measurements post-mortem were similar between all diet groups for both control and DSS-induced colitis mice. Significant differences between colon lengths in vitamin B12 deficient and vitamin B12 supplemented mice exposed to DSS were observed (two-way ANOVA, *P < 0.05, **P < 0.01, *n* = 5), values are expressed as means + SEM.

Evaluation of tissue sections from the three control study groups revealed no gross morphological differences between vitamin B12 deficient, sufficient and supplemented animals (Figure 3A). Cellular infiltration, tissue damage (Figure 3A) and an increase in Muc2 staining (Figure 3B) were more evident in colonic sections taken from animals exposed to DSS. Animals fed vitamin B12 deficient or supplemented diets exhibited colonic shortening (*P* < 0.006) following exposure to DSS (Figure 3C), whereas colonic shortening post DSS was not observed in the vitamin B12 sufficient mice.

### Cyanocobalamin Deficiency Decreases DSS-induced Colonic Tissue Damage and Alters Immune Responses

**Figure 4:**
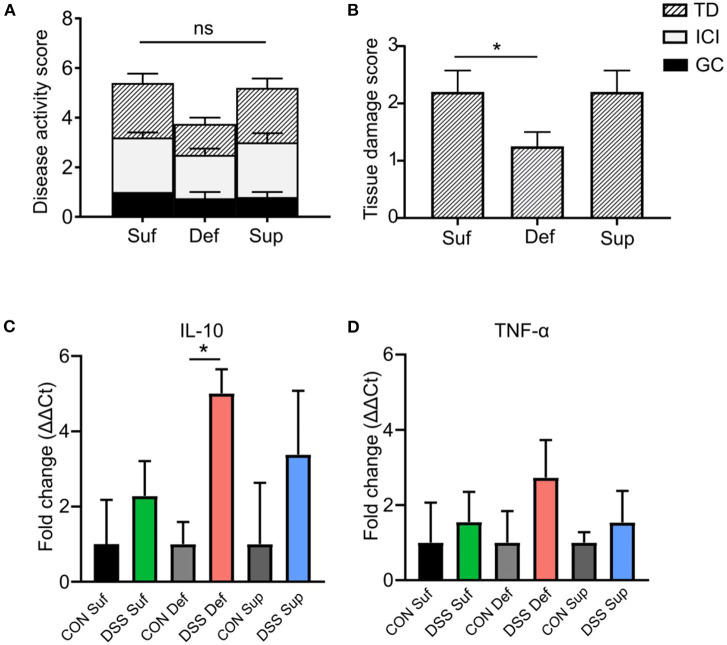
Vitamin B12 deficiency affects DSS-induced colitis tissue damage. **(A)** Composite histopathology scores of colonic tissues, graded by tissue damage (TD), inflammatory cell infiltrates (ICI) and loss of goblet cells (GC). **(B)** Tissue damage score between B12 dietary groups post DSS-treatment, B12 deficient pups show decreased tissue damage (One-way ANOVA, *P* = 0.021, *n* = 5). Normalized mRNA fold change expression ΔΔCt, between control mice and DSS treated mice. **(C)** Anti-inflammatory cytokine IL-10. Significant increase in IL-10 between B12 deficient mice post DSS compared to control (unpaired *t*-test, *n* = 3, *P* = 0.005). **(D)** Pro-inflammatory TNF-α, increased post DSS in all treatment groups but no significant difference between treatment group was found. Statisticall signigicance represented as *p < 0.05. Values are expressed as means + SEM.

As shown in Figure 4A, the composite disease activity score combining tissue damage, inflammatory cell infiltrates and goblet cell depletion, showed no significant differences among B12 diets post-DSS exposure. However, a significant decrease in epithelial tissue injury was found in mice fed a B12 deficient diet (*P* = 0.0264), compared to the other B12 treatment groups (Figure 4B). Colonic expression of anti-inflammatory cytokine IL-10 was significantly increased (4-fold; *P* = 0.005) in vitamin B12 deficient mice post DSS challenge compared to control animals (Figure 4C). Pro-inflammatory cytokine TNF-α was increased in all three-diet treatment groups following exposure to DSS; however, no statistically significant differences were observed between various dietary treatment study groups (Figure 4D).

### Altered Vitamin B12 in the Diet Does Not Affect Gut Microbial Diversity or Community Composition

**Figure 5:**
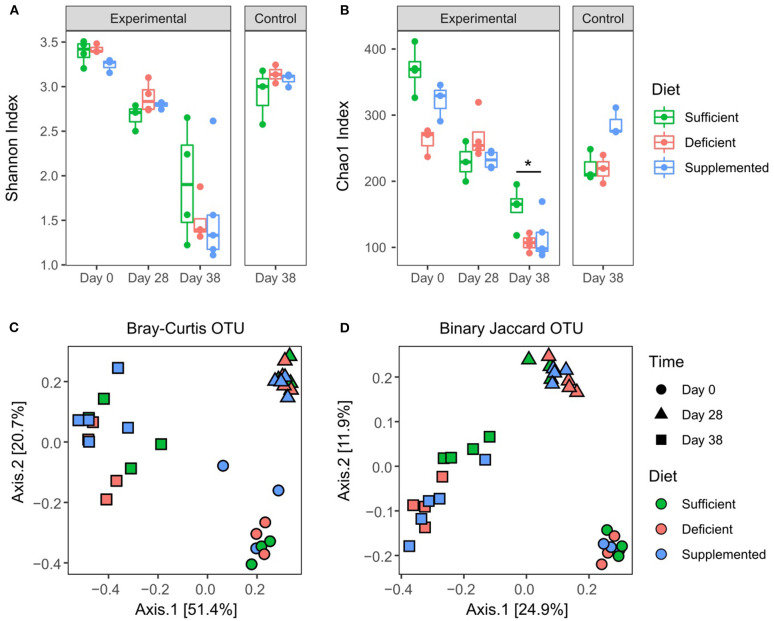
Effects of varying levels of dietary vitamin B12 on fecal microbial diversity and composition. Alpha diversity: Shannon index **(A)**, OTU table rarefied 12,000. No significant differences between dietary groups post 4 weeks of intervention, or post DSS treatment. Chao1 index **(B)**. Significant effects were found for both B12 diet (F_2, 24_ = 5.79, *P* = 0.009) and diet and time interaction (F_4, 24_ = 5.5, *P* = 0.002), Two-way ANOVA followed by Tukey's *post-hoc* test. Beta diversity: Binary Jaccard **(C)** and Bray-Curtis **(D)** were analyzed on OTU table rarefied to 12,000 reads. Significant differences were found within dietary intervention groups when comparing four weeks of dietary invention to baseline, however no differences between vitamin B12 diet intervention groups were identified at any time point. PERMANOVA *P* < 0.05, statistical singnicance represented as *p < 0.05.

Sequencing of the 16S rRNA V3 gene resulted in a total of 281,1112 reads with a minimum and maximum number of reads per sample of 9,411 and 123,598; with 2,250 assigned OTUs. Alpha diversity measured by Shannon index (Figure 5A) significantly decreased (*P* < 0.0001) in all dietary intervention groups over the four weeks prior to DSS treatment, likely reflecting compositional changes when transitioning from mother's milk to standard mouse chow (34–36). However, significant differences in alpha diversity were not observed between vitamin B12 deficient, sufficient or supplemented groups of mice after 28 days on respective diets. DSS-induced colitis resulted in a further decrease in alpha diversity in all three study groups, however no significant differences were detected in relation to vitamin B12 status. Species richness was measured by Chao1 index, which favors low abundant species. Significant variation in richness at baseline was found to normalize after 4 weeks of dietary intervention, indicating that under healthy conditions varying vitamin B12 levels do not significantly affect species richness (Figure 5B). Interestingly, mice fed a vitamin B12 sufficient diet exhibited significant retention of species richness following DSS exposure compared to B12 deficient animals (*P* = 0.03) (Figure 5B). A difference in species richness was also observed in vitamin B12 supplemented mice after 4 weeks of dietary intervention. This was indicated by an increased richness compared to both vitamin B12 deficient and B12 sufficient mice. This differential effect was observed only after 38 days of dietary intervention, likely indicating a temporal response of the gut microbiota to alterations in the availability of vitamin B12.

Overall microbial compositional changes were evaluated using two beta diversity metrics, Bray-Curtis dissimilarity (Figure 5C) and Binary Jaccard similarity index (Figure 5D). Clustering by sampling time point was observed with significant PERMANOVA (*P* = 0.001), indicating a significant shift in the gut microbiota composition after four weeks of dietary intervention, as well as a significant interaction between sampling date and diet conditions (*P* = 0.019) was found for Bray Curtis similarity. However, no significant differences in beta diversity were detected with respect to varying levels of vitamin B12 in the dietary intervention at either four weeks (day 28) or following exposure to DSS (day 38) and the induction of colitis (*P* = 0.29, *P* = 0.36 Bray Curtis and Jaccard, respectively).

### Genus Level Changes in Gut Microbiota Composition

**Figure 6:**
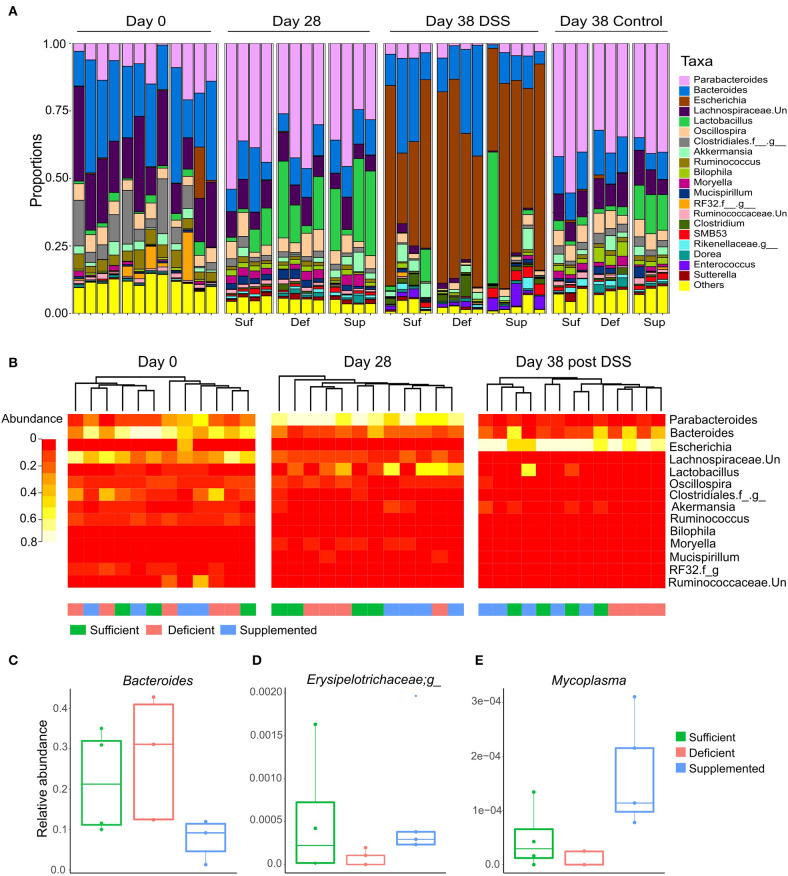
**(A)** Dominant shifts in the top 20 taxa relative abundance at the genus level in response to dietary vitamin B12 treatments and DSS-induced colitis. **(B)** Hierarchical clustering of top 25 taxa at the genus level at days 0, 28, and for DSS-induced colitis, day 38. Clustering was observed for vitamin B12 supplemented animals at day 28, which was eliminated following DSS-induced colitis. Clustering of the vitamin B12 deficient group was noted following induction of colitis at day 38. Relative abundance of taxa was significantly different between treatment groups at day 38 following DSS-induced colitis (Kruskal Wallis, FDR <0.1) **(C)** *Bacteroides*, **(D)** unclassified member of the *Erysipelotrichaceae* family, and **(E)** *Mycoplasma*.

Changes in relative abundance of the top 20 genera, revealed shifts in dominant taxa (Figure 6A). Changes in relative abundance were assessed within groups by permutation t-test between baseline and week four (day 28) and corrected for multiple comparison using FDR. No taxa were found to significantly change with FDR < 0.05, however a total of 15 taxa changed following diet treatments for four weeks (FDR < 0.1; Supplemental Table 1). The relative abundance of *Parabacteroides, Sutterella* and a member of the *Rikenellaceae* family each increased and levels of *Ruminococcus* decreased after 4 weeks, irrespective of the dietary treatment intervention.

In the vitamin B12 sufficient diet group of mice, 3 bacterial taxa uniquely changed from day 0 to 28: *Moryella* and *SMB53* exhibiting increase in abundance, and an unclassified member of the Lachnospiraceae family decreasing in abundance by more than 15% (Supplemental Table 2). Interestingly, *Bacteroides* decreased in both the vitamin B12 supplemented and deficient mice but remained unchanged in animals fed the vitamin B12 sufficient chow.

Hierarchical clustering of the top 25 bacterial genera at baseline (day 0), week 4 (day 28) and following DSS exposure (day 38) revealed clustering of the vitamin B12 supplemented group after 4 weeks of dietary intervention, and clustering of the deficient vitamin B12 group post DSS (Figure 6B). These changes indicate a partial differential response to diet intervention in the most abundant taxa.

### Cyanocobalamin Plays a Role in Determining Gut Microbiota Composition in DSS-induced Colitis

At the genus level, there were no detectable alterations in bacterial taxa following DSS-induced colitis in vitamin B12 sufficient mice (FDR > 0.05). By contrast, vitamin B12 deficient mice exhibited the most significant taxa alterations, with 39 taxa significantly altered after exposure to DSS **(**FDR <0.05; Table 1). In the vitamin B12 supplemented group, 17 bacterial genera were identified as significantly altered following exposure to DSS, 9 of which were also altered in the vitamin B12 deficient group (Table 1). *Parabacteroides* was significantly decreased in both the B12 supplemented and deficient groups, and an increase in *Escherichia* was also observed in both groups. Interestingly, *Lactobacillus* was significantly decreased in the vitamin B12 deficient treatment group (FDR <0.05), but not in the vitamin B12 supplemented group of mice. Additionally, *Bacteroides* and *Enterococcus* were increased only in animals exposed to the vitamin B12 deficient diet.

**Table 1:** Differences in genus level bacterial abundance in fecal pellets taken before and after exposure of mice to DSS (2%, wt/vol for 5 days).

**Consenus lineage**	**Deficient**	**Supplemented**	**Sufficient**
f__Porphyromonadaceae;g__ Parabacteroides	↓*↓↓*	↓*↓↓*	-
f__Lactobacillaceae;g__Lactobacillus	↓*↓↓*	-	-
f__Lachnospiraceae;Other	↓↓	↓↓	-
f__Ruminococcaceae;g__Oscillospira	↓↓	-	-
f__Deferribacteraceae;g__Mucispirillum	↓	-	-
f__Lachnospiraceae;g__Moryella	↓	-	-
o__Clostridiales;f__;g__	↓	↓	-
f__Ruminococcaceae;g__Ruminococcus	↓	-	-
f__Desulfovibrionaceae;g__Bilophila	↓	-	-
f__Lachnospiraceae;g__Shuttleworthia	↓	↓	-
f__Clostridiaceae;g__SMB53	↓	-	-
f__[Mogibacteriaceae];g__	↓	-	-
f__Coriobacteriaceae;g__Adlercreutzia	↓	-	-
f__Christensenellaceae;g__	↓	-	-
f__Lachnospiraceae;g__Clostridium	↓	-	-
f__Lachnospiraceae;g__[Ruminococcus]	↓	-	-
o__RF39;f__;g__	↓	-	-
f__Lachnospiraceae;g__	↓	-	-
f__Streptococcaceae;g__Lactococcus	↓	-	-
f__Lachnospiraceae;g__Roseburia	↓	-	-
f__Peptostreptococcaceae;g__ Tepidibacter	↓	-	-
f__Dehalobacteriaceae;g__ Dehalobacterium	↓	-	-
f__Erysipelotrichaceae;g__	↓	-	-
f__Pseudomonadaceae;g__ Pseudomonas	↓	-	-
f__Ruminococcaceae;g__Butyricicoccus	↓	↓	-
f__Peptococcaceae;g__	↓	-	-
f__Erysipelotrichaceae;g__cc_115	↓	-	-
f__Bradyrhizobiaceae;g__Bradyrhizobium	↓	↓	-
f__Lachnospiraceae;g__Blautia	↓	-	-
f__[Mogibacteriaceae];Other	↓	↓	-
f__Ruminococcaceae;g__	↓	-	-
f__Oxalobacteraceae;g__	↓	-	-
f__Oxalobacteraceae;g__ Janthinobacterium	↓	-	-
f__Peptococcaceae;g__rc4-4	↓	-	-
f__Enterobacteriaceae;g__Shigella	↑	↑	-
f__Erysipelotrichaceae;g__[Eubacterium]	↑	-	-
f__Enterococcaceae;g__Enterococcus	↑↑	-	-
f__Bacteroidaceae;g__Bacteroides	↑*↑↑*	-	-
f__Enterobacteriaceae;g__Escherichia	↑*↑↑*	↑*↑↑*	-
f__Lachnospiraceae;Other	-	↓↓	-
o__Clostridiales;f__;g__	-	↓	-
f__Ruminococcaceae;Other	-	↓	-
f__[Paraprevotellaceae];g__ Paraprevotella	-	↓	-
f__Clostridiaceae;g__Clostridium	-	↓	-
f__Xanthomonadaceae;g__Dyella	-	↓	-
f__Erysipelotrichaceae;g__Allobaculum	-	↓	-
f__Mycoplasmataceae;g__Mycoplasma	-	↑	-

*↑↑↑ > 15% change in relative abundance means, ↑↑ 4-15% change, ↑ <1% change, perm-t test FDR <0.05*.

Differences in bacterial taxa between groups of mice after exposure to DSS (at day 38 of the study) indicated that the relative abundance of *Bacteroides* (Figure 6C), an unclassified genus of Erysipelotrichaceae (Figure 6D) and *Mycoplasma* (Figure 6E) were significantly different between the dietary vitamin B12 treatment groups post DSS administration.

## Discussion

In this study, we have shown that varying cyanocobalamin levels did not result in distinct changes in the murine gut microbiota under healthy conditions but did contribute to the ability of gut microbiota to maintain compositional homeostasis following chemically-induced colitis. Our results show that vitamin B12 deficiency resulted in greater dysbiosis following intestinal injury, but the shift in dominant bacterial taxa were not related to disease activity. Rather, we found that the vitamin B12 deficient group exhibited the lowest severity of colitis, highest increase in anti-inflammatory cytokine IL-10 and significantly decreased tissue damage compared to both vitamin B12 sufficient and vitamin B12 supplemented treatment groups. Previous studies describe similar results with vitamin B deficiency (14). Mechanistically, the reduction in tissue damage in B12 deficient mice is likely attributed to an increase in IL-10, which has been previously been found to reduce colonic tissue damage in the context of DSS (37). However, the reduction in tissue-specific damage could also be a result of reduced T-cell and NK cell responses since previous work demonstrated that vitamin B12 deficiency results in decreased populations of both CD8+ cell and NK cells (38). Despite these findings several studies report lower levels of vitamin B12 in IBD patients compared with healthy controls (6). We postulate that inflammation in the distal small intestine at the site of the cobalamin receptor likely contributes to malabsorption of vitamin B12, although further studies are needed to fully understand the underlying mechanisms of action mediating the observed effects of dietary manipulations of the micronutrient.

In contrast to the observed decrease in disease severity in vitamin B12 deficient mice, we found a differential response in overall colon length following chemically induced colitis. Both deficient and supplemented vitamin B12 dietary groups exhibited colonic shortening, compared to controls whereas the sufficient vitamin B12 group of mice had no change in colon length following exposure to DSS. This finding suggests a different mechanism for vitamin B12, such as downstream metabolites generated from vitamin B12 utilization or synthesis may mediate disease severity and overall morphological changes, at least in the context of chemically induced (DSS) colitis. The observed decrease in colon length in the vitamin B12 deficient group of mice could stem from the close relationship of vitamin B12 to other micronutrients, such as folic acid. In cases of vitamin B12 deficiency, folate cycling is reduced, subsequently resulting in the overall decrease in folic acid metabolic product methylene-tetrahydrofolate, a precursor to thymidine (39). Reduction in thymidine levels impairs DNA replication and, consequently, cell proliferation, which could contribute to the observed reduction in colon length.

Many microbes colonizing the human gut encode vitamin B12-dependent genes and have sophisticated vitamin B12 transporter systems to both sense environmental levels of B12 and compete with the host for vitamin B12 utilization (40). By contrast, only a small percentage of bacteria have the capability to synthesize vitamin B12. Remarkably, members of the gut microbiota have evolved to preferentially bind various bioactive forms of cobalamin. A recent study described cyanocobalamin binding more efficiently to vitamin B12 responsive riboswitches compared to more bioavailable methylcobalamin (16). Bacteria capable of utilizing unique energy sources, such as host-associated luminal mucus, synthesize, and release vitamin B12, which subsequently can be utilized by neighboring microbial species, forming an intestinal symbiosis (41).

It has been hypothesized that interactions between vitamin B12 synthesizing and vitamin B12 utilizing microorganisms are essential for the gut microbiota to maintain compositional homeostasis (42). Such a relationship is reflected in the findings of our study, where dysbiosis observed following challenge with DSS was greater in the vitamin B12 deficient group of mice, compared to the two other dietary intervention study groups. Following experimental colitis, vitamin B12 deficient mice exhibited 30 bacterial taxa that were uniquely changed, with 39 taxa significantly altered in total. Within these altered taxa, an increase of opportunistic enteropathogens was observed, including *Shigella* and *Enterococcus*. Despite this increase in pathogenic bacteria, we observed a decrease in colonic tissue damage, indicating that vitamin B12 deficiency may have a greater impact on epithelial regeneration and inflammation-induced damage than effects on microbial composition.

Interestingly, sufficient vitamin B12 dietary treatment group exhibited the most resistance to microbial dysbiosis following DSS-induced colitis (no bacterial taxa were significantly altered post DSS treatment), while the B12 supplemented group exhibited a moderate microbiota response.

The vitamin B12 sufficient group of mice also retained more species richness following exposure to DSS, as compared to either deficient or supplemented mice; findings which support the role of B12 in maintaining gut microbiota homeostasis. Of the 3 bacterial taxa found to differentially respond to DSS-induced colitis between vitamin B12 dietary groups, 2 identified at the genus level (*Bacteroides* and *Mycoplasma*) are known to contain vitamin B12 processing genes (40). *Bacteroides* genus has the most well-studied vitamin B12 capture, transport and utilization systems, and possess an additional protein, BtuG2, that allows the microorganism to outcompete the host for binding to vitamin B12 (43). Previous work demonstrated that supplementation of vitamin B12 selectively downregulates *Bacteroides* abundance, whereas we observed a decrease in abundance in vitamin B12 deficient mice as well as supplemented mice (44). Notably, *Bacteroides* abundance significantly increased post DSS treatment in B12 deficient mice while remaining unchanged in B12 supplemented mice. *Bacteroides* genus competition for vitamin B12 also influences the virulence of pathogenic *E. coli* (45), suggesting that inter-bacterial competition for vitamin B12 is likely to play a key role in balancing the gut microbial ecosystem (42). Vitamin B12 supplementation enhances the production of Shiga toxin-2 by enterohemorrhagic *E. coli* O157:H7 (45), through the ethanolamine utilization (*eut*) operon. This virulence factor regulator is common among enteric bacterial pathogens such as *Salmonella* (46), *Clostridium* (47), and *Enterococcus* (48). Thus, in the setting of vitamin B12 deficiency regulation of such virulence factors in bacteria could be a mechanism underlying the improvement of tissue damage in murine models of colitis.

## Limitations and Conclusions

These results provide valuable insights into the interactions between vitamin B12 and the gut microbiota, both in the absence and presence of intestinal inflammation. However, there are a few limitations that should be considered. Firstly, the use of DSS as a model of colitis is but one of many models of IBD (49). Future studies should, therefore, determine whether vitamin B12 status affects disease outcomes and microbiota composition in other well-established models of colitis (such as interleukin-10 gene knockout mice). Secondly, local inflammatory markers in this study were limited to IL-10 and TNF-α. Additional studies evaluating additional immune responses will help delineate the effects of vitamin B12 status in the setting of health and disease. Neutrophil-derived myeloperoxidase has been shown to increase in response to DSS-induced colitis (50) and would prove beneficial in correlating inflammatory responses to vitamin B12 status. Thirdly, the use of synthetic cyanocobalamin as the only form of vitamin B12 did not allow for an analysis of potential differential effects of alternative bioactive forms of the vitamin. The addition of other forms of cobalamin such as methylcobalamin along with measurement of cobalamin levels in the intestinal lumen would allow for a better understanding of how cobalamin status impacts mucosal inflammation and microbiota homeostasis. Lastly, the use of only female mice limited our ability to delineate sex-related differences. The addition of metagenomic sequencing and transcriptomics would be a logical next step to evaluate the role of vitamin B12 under conditions of dysbiosis in the pathogenicity and severity of experimental colitis.

In conclusion, these results demonstrate that altered dietary levels of vitamin B12 under conditions of intestinal health do not significantly affect the gut microbial composition. By contrast, following experimental colitis, vitamin B12 deficiency decreases colonic tissue damage, but results in greater intestinal microbial dysbiosis, and promotes the growth of opportunistic pathogens.

## Data Availability Statement

Sequence data have been submitted to the European Nucleotide Archive (ENA) and can be accessed under study accession PRJEB37474.

## Ethics Statement

The animal study was reviewed and approved by Hospital for Sick Children Laboratory Animal Services (Protocol# 37209).

## Author Contributions

EL designed the research. EL, BL, PM, and RW conducted the experiments. LR and MS performed the 16S rRNA gene sequencing. Data analysis and interpretations were done by RH, EL, SB, and BL. RH, KJ-H, and PS wrote the manuscript and all authors revised and approved the final version of the manuscript.

### Conflict of Interest

PS serves on advisory boards for Antibe Therapeutics, Cargill and Nestle-Gerber. He has received honoraria from Abbott Nutrition for presenting at continuing medical education events. The remaining authors declare that the research was conducted in the absence of any commercial or financial relationships that could be construed as a potential conflict of interest.
