# Supplementary material for: Vitamin B12 Deficiency Alters the Gut Microbiota in a Murine Model of Colitis
Source: Front Nutr. 2020 Jun 5;7:83. doi: 10.3389/fnut.2020.00083 (PMC7291859; doi:10.3389/fnut.2020.00083)
Supplement: Supplementary file 1 [file Data_Sheet_1.docx]

**Supplemental Material**

**Supplemental Table 1. Primer sequences used in RT-qPCR.**

| Target | Forward | Reverse |
| --- | --- | --- |
| IL10 | 5’-GCTCTTACTGACTGGCATGAG-3’ | 5’-CGCAGCTCTAGGAGCATGTG-3’ |
| TNFa | 5’-CCCTCACACTCAGATCATCTTCT-3’ | 5’-GCTACGACGTGGGCTACAG-3’ |
| RPL-10 | 5’-GTGATTGAGGCTCTGCGAAGAG-3’ | 5’-ATGAGCCGCTTCTCAGCAACCA-3’ |
| GAPDH | 5’-AGGTCGGTGTGAACGGATTTG-3’ | 5’-TGTAGACCATGTAGTTGAGGTCA-3’ |

**Supplemental Table 2. Significant differences in genus level relative abundance taxa after 4 weeks of vitamin B12 diet treatment.**

| Consenus Lineage | Deficient | Supplemented | Sufficient |
| --- | --- | --- | --- |
| f__Porphyromonadaceae;g__Parabacteroides | ↑↑↑ | ↑↑↑ | ↑↑↑ |
| f__Bacteroidaceae;g__Bacteroides | ↓↓ | ↓↓↓ | - |
| f__Desulfovibrionaceae;g__Bilophila | ↑ | - | ↑ |
| f__Ruminococcaceae;g__Ruminococcus | ↓ | ↓ | ↓ |
| o__RF32;f__;g__ | ↓ | - | - |
| f__Deferribacteraceae;g__Mucispirillum | ↑ | - | ↑ |
| o__Clostridiales;f__;g__ | ↓↓ | - | - |
| f__[Mogibacteriaceae];g__ | ↑ | ↑ | ↑ |
| f__Alcaligenaceae;g__Sutterella | ↑ | ↑ | ↑ |
| f__[Paraprevotellaceae];g__Paraprevotella | ↓ | ↓ | - |
| f__Erysipelotrichaceae;g__Clostridium | ↑ | - | ↑ |
| f__Rikenellaceae;g__ | ↑ | ↑ | ↑ |
| f__Erysipelotrichaceae;g__[Eubacterium] | - | ↑ | - |
| f__Lachnospiraceae;Other | - | - | ↓↓↓ |
| f__Lachnospiraceae;g__Moryella | - | - | ↑ |
| f__Clostridiaceae;g__SMB53 | - | - | ↑ |

↑↑↑ > 15% change in relative abundance means, ↑↑ 4-15% change, ↑ <1% change, perm-t test FDR<0.10
